# Beyond correlation and causation: epistemic caution, anomalous findings, and COVID-19 vaccine safety

**DOI:** 10.1186/s13010-026-00232-3

**Published:** 2026-10-01

**Authors:** Alex S. Siebner, Alberto Rubio-Casillas, Mikolaj Raszek, David Cowley, Mark Fabrowski, Marina Piscopo, Carlo Brogna, Vladimir N. Uversky, Elrashdy M. Redwan, Sylvia N. Genova

**Affiliations:** 1 Institute for Tropical Medicine, University of Tübingen, Tübingen, Germany; 2 German Center for Infection Research, Partner Site Tübingen, Tübingen, Germany; 3 Jalisco Health Services, Autlan Regional Hospital, Jalisco, Autlan 48900 Mexico; 4 Biology Laboratory, Autlan Regional Preparatory School, University of Guadalajara, Guadalajara, Mexico; 5 AB T5J 3R8, Merogenomics (Science Content Development and Education), Edmonton, Canada; 6 Independent Researcher, Nottingham, UK; 7 Department of Emergency Medicine, Eastern Road, Royal Sussex County Hospital, Brighton, UK; 8 The Tick Borne Disease Clinic, West Sussex, UK, https://www.tbdclinic.com/; 9 Department of Biology, University of Naples Federico II, Naples, Italy; 10 Department of Research, Craniomed Group Facility Srl., Montemiletto, Italy; 11 Department of Molecular Medicine and USF Health Byrd Alzheimer’s Research Institute, University of South Florida, Tampa, USA; 12 Department of Biological Sciences, King Abdulaziz University, Jeddah, Saudi Arabia; 13 Therapeutic and Protective Proteins Laboratory, Protein Research Department, Genetic Engineering and Biotechnology Research Institute, City of Scientific Research and Technological Applications, Alexandria, Egypt; 14 Department of General and Clinical Pathology, Medical University Plovdiv, Plovdiv, Bulgaria; 15 Morphological Research Institute, Medical University Plovdiv, Plovdiv, Bulgaria

**Keywords:** Scientific paradigm, Vaccine safety, COVID-19 vaccines, Severe adverse events, Correlation-causation, Bradford Hill considerations

## Abstract

The COVID-19 pandemic exposed science’s adaptive strengths and epistemic limits. In fact, in the pandemic, mRNA vaccines are attributed to have saved millions of lives. The maxim “correlation does not imply causation” is an essential safeguard against premature causal inference. However, its use may become epistemically restrictive when the absence of established causality is treated as sufficient reason not to investigate (severe) vaccine adverse events. This article examines the conditions under which a valid cautionary principle may have contributed to premature epistemic closure. Thomas Kuhn’s theory of scientific paradigms is used as one interpretative lens rather than as an empirical explanation of institutional behaviour. We argue that the correlation–causation distinction can shift from a heuristic of caution to an instrument of epistemic conservatism, reinforcing prevailing frameworks and discouraging exploration of anomalies. A published autopsy case report involving multifocal necrotizing encephalitis and myocarditis after BNT162b2 vaccination is examined as an illustrative and hypothesis-generating case, not as representative evidence of systematic institutional conduct. A qualitative approach using Bradford Hill’s criteria provides partial, inconclusive evidence of a causal relationship while also exposing the methodological limits of the tool employed. The results of the analysis showed that one of the nine criteria demonstrated support of causality, seven were partially supported, and one did not support causality. The analysis suggests, but does not prove, a causal relationship between the BNT162b2 vaccine and multifocal necrotizing encephalitis. The absence of the nucleocapsid protein does not provide definitive evidence to demonstrate a vaccine origin. Analytical methods are described which can distinguish between the wild-type SARS-CoV-2 spike protein and the recombinant spike protein expressed following mRNA vaccination. The absence of evidence does not mean evidence of absence and we must keep an open mind to this when reviewing such evidence on causality.

## Introduction

The COVID-19 pandemic clearly demonstrated the capacity for rapid behavioural adaptation across public and private life and scientific practice. Social interactions were minimized, physical distancing was instituted, and mask-wearing became the standard practice. These changes were implemented not only as temporary crisis measures but as part of a broader reconfiguration of societal norms, informed by evolving epidemiological evidence and public health guidelines. Adenoviral- and mRNA-based vaccines became a major public health intervention, and health authorities asserted their safety and efficacy. Pharmacovigilance was monitored during the rollout of the vaccines. The presented article does not generally assume that vaccine-safety communication constituted a monolithic paradigm or that scientists were systematically pressured to conform. For that, it can only give circumstantial evidence. The article cautiously asks a narrower question of whether, in some circumstances, strong institutional reassurance and high evidentiary thresholds can make anomalous safety observations more difficult to investigate and to communicate.

The newly established mRNA vaccines are estimated to have saved 14–20 million lives worldwide in the first year of rollout, according to the World Health Organization [1]. However, a more conservative analysis suggests a significantly lower number, limited to older individuals, ranging from 1 to 4 million between 2020 and 2024 [2]. At the post-authorization surveillance level, some concerns have been raised about vaccine safety for COVID-19 vaccines, particularly mRNA vaccines, as some published literature has suggested a possible link to excess mortality. Paradoxically, despite Omicron’s milder disease profile, countries with extensive vaccination coverage registered unanticipated increases in excess mortality during and after the Omicron waves [3–12]. The temporal association between widespread COVID-19 vaccination campaigns and concurrent changes in excess mortality patterns requires rigorous systematic investigation.

“Correlation does not imply causation” is a fundamental concept in epidemiology and statistics because observed associations may reflect confounding, bias, selection processes, measurement error, or chance rather than a direct causal relationship [13, 14]. Although the maxim is essential for avoiding premature conclusions, it can be misused, leading to the premature decision not to initiate investigations into possible causal relationships [13]. Thomas S. Kuhn’s (1922–1996) framework of scientific change, shown in his seminal book *The Structure of Scientific Revolutions* (1962), questioned the belief that science develops only through the steady accumulation of empirical data. It is used here as a conceptual and interpretive lens [15]. He observed that scientific communities generally resist anomalous findings that challenge the prevailing paradigm. By itself, it cannot demonstrate that a particular institution, regulator, journal, or scientific community intentionally acted to protect a prevailing paradigm. This resistance to accepting a new framework can lead to the premature dismissal of emerging causal hypotheses if they are incompatible with the accepted theoretical framework [15]. Instead, he argued that scientific progress occurs within paradigms: shared frameworks of theory, methodology, and underlying assumptions that determine what questions scientists consider legitimate, what evidence they deem valid, and what explanations they find acceptable [15]. A paradigm, according to Kuhn, defines not only the answers but also the very structure of permissible research. Scientists operating within a paradigm are engaged in what he termed “normal science”, a process of puzzle-solving within the boundaries of accepted theory. Findings that defy the conventional paradigm are referred to as “anomalies”. Only when these anomalies accumulate to an inflection point does the prevailing paradigm enter a “crisis phase”, which can potentially lead to a paradigm shift and a radical restructuring of the conceptual framework [15]. Kuhn’s framework is fundamentally a philosophical account of scientific change and one of several theories that can be applied.

During the COVID-19 pandemic, public and scientific communications frequently distinguished temporal reports of severe adverse events (SAEs) from (severe) adverse events (AEs) for which causality has been established. Some SAEs were disregarded on the basis that a correlation did not prove causation.

This distinction is theoretically valid but could have prevented further research by discouraging rigorous empirical inquiry. Reports of temporally associated SAEs, such as autoimmune reactions, neurological syndromes, cardiovascular events, or sudden death, were labelled as ’coincidental’ without thorough studies being performed [16]. As the evidence base has expanded, researchers with differing perspectives have argued that certain safety signals warrant closer examination [17]. Whether these observations reflect causal associations or residual confounding remains the subject of ongoing scientific investigation, highlighting the iterative nature of pharmacovigilance and causal inference. Temporal association is not itself sufficient to establish causation, but neither is it irrelevant. Safety surveillance commonly compares the observed frequency of AEs with the frequency expected in a background population. A recent review describes observed-to-expected analyses as a quantitative signal-detection tool used during COVID-19 vaccine surveillance from an industry perspective. It emphasizes their methodological limitations and the need to interpret them alongside case reviews, trend analyses, epidemiological studies, and the wider literature [18]. If the confirmed rate of an illness is greater than the expected background rate, it triggers a safety investigation. In terms of reaching a conclusion on causality, biological plausibility, consistency and alternative explanations are checked with the WHO Causality Framework. The WHO defines a vaccine-safety signal as information suggesting a new or incompletely documented potentially causal association between vaccination and an AE. Very rare events remain particularly difficult to identify and may require large, linked, multinational datasets [19].

The principle that “correlation does not imply causation” or “causality has not been established” may be interpreted as epistemically closing a question when it is not accompanied by an explanation of remaining uncertainty, the evidentiary requirements for causal evaluation, or plans for further investigation.

This article does not establish if temporally associated SAEs were systematically dismissed, with questions in this regard remaining not fully elucidated. Rather, it delves into the conditions under which a valid cautionary maxim can be employed in a way that discourages the generation of follow-up research hypotheses. It advances a limited normative claim: the statement that correlation does not establish causation should ordinarily initiate, rather than terminate, consideration of whether further causal investigation is warranted. It cannot empirically demonstrate that vaccine-safety surveillance was systematically suppressed during the COVID-19 pandemic.

Here, we analyse a single case autopsy report as an illustrative case that introduced an immunohistochemical (IHC) method that supposedly could demonstrate whether mRNA-based vaccines against SARS-CoV-2 contributed to or were the cause of death [20]. It can show how temporal, pathological, and molecular observations may generate a causal hypothesis while remaining insufficient to establish individual causation. The case is therefore used to clarify evidentiary limits, not to infer the behaviour of the whole scientific community.

## The correlation-causation distinction: origins and utility

The differentiation between correlation and causation is essential in medical research. Correlation does not necessarily mean that changes in one variable result in changes in the other. This misunderstanding may lead to inappropriate clinical judgments and public health policy [13, 14, 21]. David Hume (1711–1766), a preeminent philosopher of the 18th century, reshaped the philosophical understanding of causation through his rigorous analysis of causal inference. He suggested that the human tendency to perceive causal connections is rooted not in logical necessity or direct observation, but rather in psychological habit and expectation [22]. In the 20th century, statisticians such as Austin Bradford Hill (1897–1991) and Ronald A. Fisher (1890–1962) developed rigorous methodologies to evaluate whether correlations in observational data could indicate causal relationships, especially in epidemiology [23]. Their contributions ranged from epidemiological associations between smoking and lung cancer, neurodevelopmental impairments attributable to lead exposure, and the mortality risk associated with air pollution. Notably, in each case, correlation served as the foundation for further investigation rather than a reason to dismiss it [23].

### Plural approaches to scientific change and causal inference

Kuhn’s philosophical framework is not the only one that disputes over anomalous evidence. Popper emphasizes critical testing and potential falsification of hypotheses. They are true until the opposite has been demonstrated [24]. Lakatos developed this concept further and distinguished progressive from degenerative research programmes [25]; Longino located scientific objectivity in appropriately structured critical interactions within the scientific community [26]; and Kitcher analysed scientific progress through both epistemic and institutional practices connected to priorities and resources [27].

These perspectives differ in their accounts of evidence, criticism, consensus, and scientific institutions. Here, Kuhn is retained because the concepts of normal and abnormal science are particularly useful for the present discussion. We do not claim that the Kuhnian account is uniquely correct.

Contemporary causal inference also extends substantially beyond a correlation–causation maxim and Bradford Hill considerations. For example, potential-outcomes approaches define causal effects through comparisons of alternative exposure states, which would allow a broader perspective; structural causal models make causal assumptions explicit, interventionist theories examine whether manipulating one variable would change another, and causal pluralist approaches emphasize that epidemiological, experimental, and contextual evidence answer different causal questions [28–31]. A pathological observation in a case report can contribute to hypothesis generation and to assessing mechanistic plausibility. It cannot substitute for population-level effect estimation, source-specific molecular attribution, or controlled intervention.

## Vaccine safety, pharmacovigilance, and the institutional context of causal evaluation

Vaccination has played a critical role in reducing infectious disease morbidity and mortality rates, helping to meet Sustainable Development Goal 3, which aims to guarantee healthy lives and promote well-being at all ages [32–34]. By reducing uneven access to healthcare and economic disparities among various socioeconomic groups, vaccines are largely regarded as one of the most effective strategies and as being crucial to advancing global health [35]. As demonstrated in several reports, specific vaccines are highly effective in preventing child mortality from several diseases, including beneficial non-specific effects [32, 36–42]. Health authorities must rigorously assess the benefits of vaccination against potential risks when addressing diseases with significant morbidity and mortality. While vaccines can cause AEs and induce theoretically unknown risks, the full safety profile only emerges after widespread population use. These potential harms can complicate vaccination decisions at both individual and policy levels, making comprehensive risk assessment challenging until extensive real-world deployment occurs [43].

During COVID-19 vaccination, emerging vaccine safety signals, such as myocarditis, thrombotic events, anaphylaxis, myocardial infarction, pulmonary embolism, or death, were reported [44, 45]. These reports of events occurring after vaccination are treated in pharmacovigilance initially as signals rather than established adverse reactions. The statement that causality has not been demonstrated can serve a legitimate methodological function to distinguish temporal sequence from causality. The epistemic concern arises only when the statement is treated as sufficient to end surveillance; controlled epidemiology, pathology, or experimental work is warranted.

The present analysis distinguishes institutional intention from communicative effects. Public health authorities, regulatory agencies, and media outlets may unintentionally reduce the perceived importance of anecdotal or post-marketing reports, particularly when uncertainty and the requirements for subsequent causal assessment are not made explicit. This work cannot prove that authorities intended to protect a vaccine program or to delegitimize safety reports. Nevertheless, vaccination mandates and vaccination-status restrictions generated substantial opposition in several countries [46]. Research indicates that public debate often blurred the distinction between general opposition to vaccination and opposition specifically to compulsory vaccination, while unvaccinated individuals experienced moral reproach, polarization, and, in some settings, support for unequal or exclusionary treatment [47–49].

(i) Sociology of science provides reasons to examine how institutional structures can influence the reception of controversial findings or hypotheses without assuming deliberate censorship. Empirical and theoretical work describes tensions between norms of organized scepticism and counter-norms involving commitment, authority, reputation, and disciplinary boundary work [50–53]. Public interviews with vaccine critics can be analysed as primary discourse material to investigate how dissenting actors framed expertise, institutional trust, uncertainty, and perceived exclusion, but they should be treated as objects of discourse analysis rather than as evidence for biomedical causality [54–57]. These frameworks support the possibility that hierarchy, institutional affiliation, and accepted evidence standards influence which claims receive attention.
(ii) Demonstrating systematic institutional discouragement would require a separate empirical study, such as a preregistered content analysis of regulatory communications, comparisons of funding and editorial decisions, examination of internal policy documents, or interviews with researchers and regulators. Because the present article does not undertake such analyses, institutional suppression and paradigm protection are treated as hypotheses for future investigation rather than as demonstrated findings. Therefore, this article can explore circumstantial evidence for editorial decisions during the COVID-19 pandemic. Interestingly, empirical studies of scientific publishing predominantly showed accelerated processing of COVID-19 manuscripts rather than delayed editorial decisions. Non-COVID-19 manuscripts were delayed in comparison [58–63]. This does not establish that dissenting or vaccine-safety manuscripts were selectively delayed. But it could show that timely pressure was generated to publish everything related to COVID-19 – potentially resulting in less careful editorial and reviewer decisions. Testing this hypothesis would require data for both accepted and rejected submissions within editorial timelines, reviewer invitations, decision letters, and all manuscript characteristics. Another approach would be to assess preprints related to COVID-19 and their time to peer-reviewed publication. Of special interest would be whether critical, unfavourable, or safety-concerning findings remained longer as preprints than others or were less likely to lead to peer-reviewed publication. Several studies assessed COVID-19 preprint publication rates and lag times. These studies have documented variation in the probability and timing of journal publication following COVID-19 preprints [64–66]. One study found that COVID-19 articles were also more likely to be retracted [65]; another that larger sample size and low risk of bias (assessed by study authors via a risk of bias tool) were associated with journal publication [67]. They did not assess whether the results were critical of vaccination – acknowledging a scientific gap.

In Germany, the Paul-Ehrlich-Institut conducted a large prospective SafeVac 2.0 study to actively monitor the safety and tolerability of COVID-19 vaccines [68]. Although follow-up was scheduled to conclude in 2023 and selected analyses have been released [69], a comprehensive peer-reviewed publication of the complete study results could not be identified as of July 2026.

The statement from Carl Sagan, “absence of evidence is not evidence of absence” [70], is only a philosophical principle and could be relevant when available studies are underpowered, insensitive, or not designed to detect the event in question. It is not universally applicable: a well-designed study with adequate statistical power, sensitive and adequate measurements, appropriate controls, and sufficiently narrow confidence intervals may provide meaningful evidence against an effect of a specific magnitude [71]. The epistemic significance of a null result therefore depends on the design and sensitivity of the investigations. A narrower conclusion of this section is that scientific and regulatory standards shape how safety signals are classified and prioritized. A Kuhnian interpretation may illuminate this process further, but uncertainty, resource constraints, evidentiary thresholds, and precautionary communication provide plausible alternative explanations. Claims about systematic suppression require evidence beyond the philosophical framework used here. For sure, this rhetorical strategy served two functions:

(i) It protects the vaccine program from reputational harm.
(ii) It delegitimizes anecdotal or post-marketing reports as “anti-vaccine” or “misinformation”.

Other interests are financial or political. According to Kuhn, when anomalies arise, they are often initially overlooked or excluded because they do not fit within the established paradigm, which inherently limits what is observed and reported [15]. According to Kuhn, paradigms influence the questions scientists formulate, the approaches they adopt to address those issues, the standards for valid evidence, and the interpretation of findings. Scientists work within this framework, focusing on solving puzzles defined by the accepted paradigm [15]. Unfortunately, the prevailing paradigm surrounding the vaccination of people against SARS-CoV-2, which asserts the efficacy of vaccines in preventing severe illness and death, has become a dogma that precludes consideration of the possibility that vaccines may also be associated with SAEs.

## Illustrative appraisal using the Bradford Hill considerations of a single case report

In 1965, Austin Bradford Hill presented nine “viewpoints” to consider when evaluating whether an observed association might be causal: temporality, plausibility, coherence, specificity, biological gradient, analogy, consistency, experimental evidence, and strength [72]. Hill did not propose a checklist, an additive score, or a numerical probability of causation. Despite recognized limitations, particularly in precision and subjectivity, and the emergence of newer alternatives, Hill’s criteria have stood the test of time and are still widely used as a conceptual guide, especially in public health [28, 73]. The considerations are not equally informative, and failure to satisfy one or more does not automatically exclude causation. Temporality is logically necessary, whereas the relevance of the other considerations depends on the causal question, study design, and available evidence [74, 75].

In 2022, Dr. Michael Mörz published the case report *“*Multifocal Necrotizing Encephalitis and Myocarditis after BNT162b2 mRNA vaccination against COVID-19” [20]. Here, we use this report as an illustrative and hypothesis-generating case rather than as representative evidence of population-level risk or institutional conduct.

**Fig. 1:**
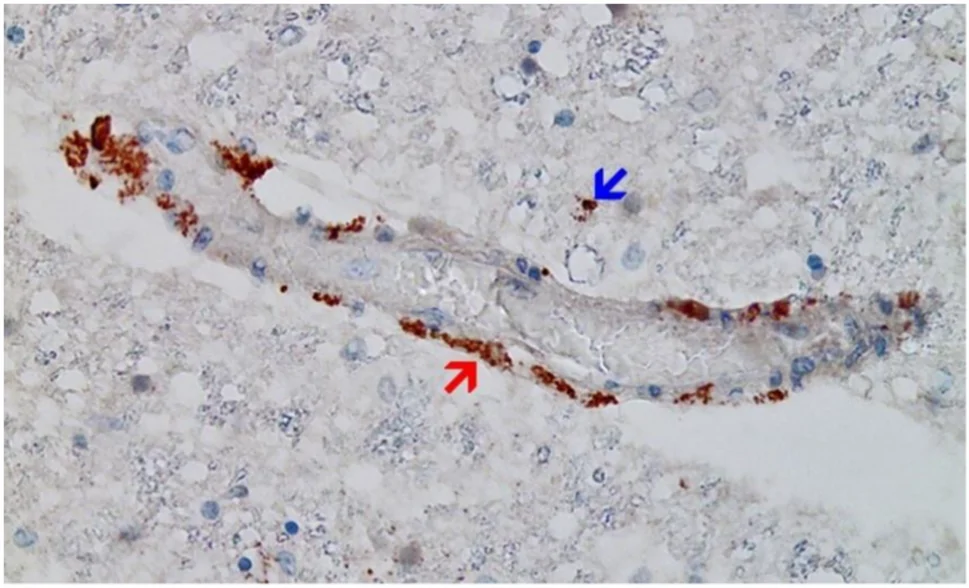
Brain, nucleus ruber. The substantial presence of the SARS-CoV-2 S protein in the congested endothelium of a capillary vessel indicates signs of acute inflammation, with sporadic mononuclear inflammatory cell infiltrates. IHC staining for the SARS-CoV-2 S protein subunit 1 revealed the presence of the antigen in the form of brown granules in capillary endothelial cells (red arrow) and individual glial cells (blue arrow). 200 × magnification. Source: [20]. This figure is open access and is distributed under the Creative Commons Attribution Non-Commercial (CC BY-NC 4.0) license

**Fig. 2:**
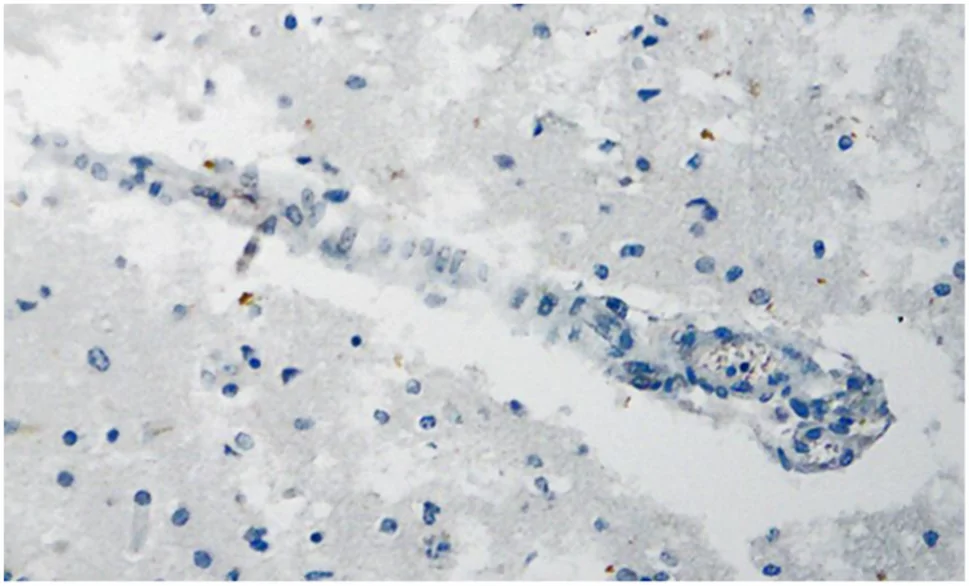
Brain, nucleus ruber. A negative IHC reaction was observed for the SARS-CoV-2 N protein. Cross-section through a capillary vessel (same vessel as shown in Fig. 1). 200 × magnification. Source: [20]. This figure is open access and is distributed under the Creative Commons Attribution Non-Commercial (CC BY-NC 4.0) license

**Fig. 3:**
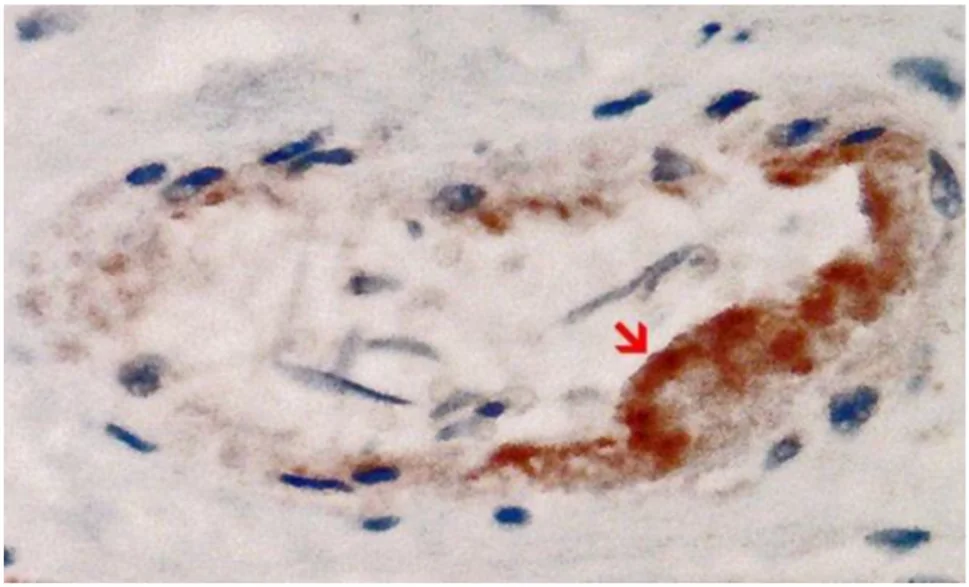
Left ventricle of the heart. A positive reaction to the SARS-CoV-2 S protein. This is a cross-section through a capillary vessel. The brown granules are the IHC representation of SARS-CoV-2 S subunit 1. A widespread distribution of the S protein in capillary endothelial cells (red arrow) is shown, which correlates with noticeable endothelial swelling and the presence of a small number of mononuclear inflammatory cells. 400 x magnification. Source: [20]. This figure is open access and is distributed under the Creative Commons Attribution Non-Commercial (CC BY-NC 4.0) license

**Fig. 4:**
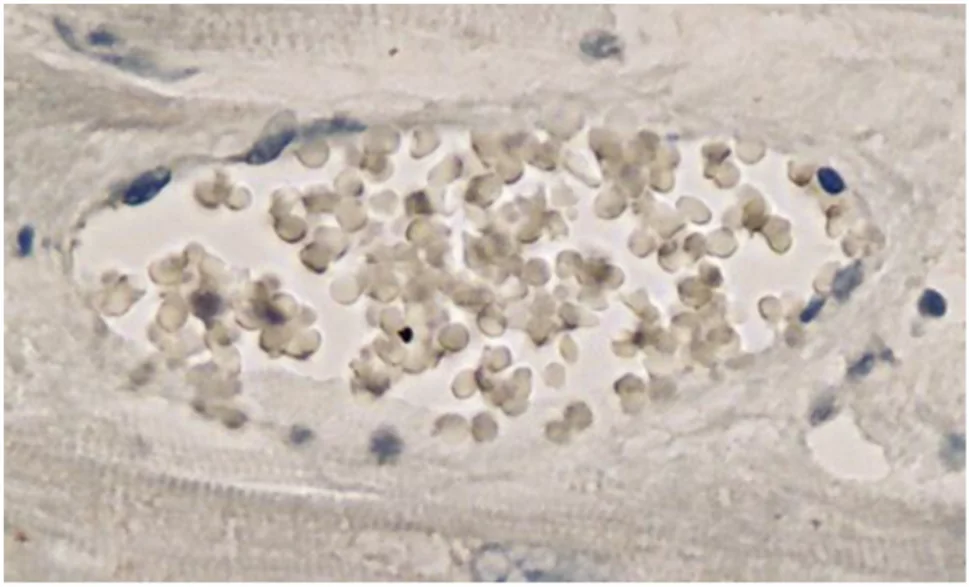
Left ventricle of the heart. A cross-section through a capillary vessel is presented. A negative immunohistochemical reaction was observed for the SARS-CoV-2 N protein (same vessel as shown in Fig. 3). 400 × magnification: Source: [20]. This figure is open access and is distributed under the Creative Commons Attribution Non-Commercial (CC BY-NC 4.0) license

We will evaluate it through the lens of the Bradford Hill considerations. Mörz reported the case of a 76-year-old male patient diagnosed with Parkinson’s disease (PD) who died 21 days after receiving a third COVID-19 vaccine. He was immunized with two doses of the mRNA-based BNT162b2 vaccine in July and December 2021, following the initial administration of the ChAdOx1 nCoV-19 vector vaccine in May 2021. Because the clinical symptoms were unclear before death, the family requested an autopsy. Post-mortem investigations confirmed PD. Additionally, there were clear signs of systemic arteriosclerosis and aspiration pneumonia. Histopathological examinations of the brain identified previously uncovered abnormalities, such as multifocal necrotizing encephalitis of undetermined aetiology, characterised by severe inflammation and a glial and lymphocytic response. There was also evidence of acute vasculitis, which was primarily lymphocytic, but no evidence of mild acute lymphohistiocytic myocarditis and vasculitis in the heart, alongside chronic cardiomyopathy. No laboratory-confirmed SARS-CoV-2 infection was documented, although this does not exclude an unrecognized or asymptomatic infection. Several studies have shown persistent shedding of SARS-CoV-2 RNA in stool, after clearance of respiratory infection [76, 77]. IHC was used to examine the nucleocapsid (N) and spike proteins (S) of SARS-CoV-2. Interestingly, it revealed anti-S, but not anti-N immunoreactivity within inflammatory areas of the brain and heart, and, remarkably, in the endothelial cells of small blood vessels. S and N proteins are expressed during SARS-CoV-2 infection, whereas vaccines (mRNA- or vector-based) encode only the S protein. The original case report interpreted the presence of the S protein and the absence of the N protein as supporting a vaccine origin. This pattern is shown in the nucleus ruber in the brain (Figs. 1 and 2) and the left ventricle of the heart (Figs. 3 and 4**)** [20].

The official cause of death was recorded as chronic aspiration-related pneumonia, a recognized complication of PD [78, 79]. Necrotizing encephalitis and myocarditis were additional findings, and the encephalitic lesions may have contributed to the clinical course. While the histopathological signs of myocarditis were relatively modest, the encephalitis had resulted in significant multifocal necrosis and may have contributed to the fatal consequence. Encephalitis frequently results in epileptic seizures, and the tongue bite observed during the autopsy indicated that this may have occurred in this instance [20]. This appraisal is deliberately limited. A single case report cannot estimate an exposure–outcome association, demonstrate consistency across independent populations, establish a population-level dose–response relationship, or quantify the strength of association; however, it can be used to evaluate temporal, pathological, and potentially mechanistic observations that justify hypothesis generation. Accordingly, we analyse this single case study to determine which of the categories can be described as *support present*, *limited support*, *not established*, or *not assessable*.

### Temporality

SAEs such as neurological and cardiac events occurred following the administration of the second and third doses of the Pfizer-BioNTech (BNT162b2) vaccine. The last dose (BNT162b2) was administered three weeks before death, and the timeline of symptom progression has been documented.

Assessment: Support present.

### Plausibility

The aforementioned temporal relationship and localization of the S protein within affected tissues without the N protein found within the brain (including endothelial cells, glial cells, and microglia) and heart was proposed to be consistent with vaccine-derived S protein rather than natural infection [20]. It provides a hypothesis-generating observation. It has been demonstrated that trace amounts of vaccine-encoded S protein could persist for several weeks [80], potentially triggering vascular inflammation and tissue damage, a mechanism that is both biologically plausible and supported by existing literature [81–83]. A mechanistic study by Schreiber et al. investigated whether the SARS-CoV-2 S1 protein by itself can affect the brain. In mice, intravenous, intranasal, and intracerebral administration of S1 triggered neuroinflammation and alterations in α-synuclein levels in regions linked to PD. The effects varied by exposure route and sex, with intravenous S1 causing the strongest α-synuclein accumulation and microgliosis. The findings suggest that circulating or tissue-retained S protein may contribute to long-term neurological changes, even in the absence of active viral infection [84, 85]. Failure to perform aspiration during the administration of mRNA vaccines in humans could potentially lead to the unintended injection of the vaccine into the bloodstream [86]. Brogna et al. described a proteomic approach intended to distinguish prefusion-stabilized vaccine-encoded S from wild-type viral S [80], which may improve source attribution but does not independently demonstrate that the detected protein caused a pathological lesion.

The reason why vaccinal S protein could be observed in the blood of patients for up to 6 months remains unclear. One hypothesis proposed by the authors was the potential involvement of the blood microbiome, where certain microbial species could theoretically have been transfected by vaccine-derived mRNA, thereby contributing to prolonged S protein detection [80]. Another potential explanation, not previously considered in this context, is that the observed S protein may have been associated with fibrinaloid microclots. Unusual, breakdown-resistant clots composed of amyloid fibrin/fibrinogen structures have been described in patients with COVID-19 [16, 87, 88] and long COVID [89–91]. Independent research groups have proposed that S protein may act as one potential trigger for fibrinaloid microclot formation, with these structures capable of entrapping the S protein in the process [92–94]. Additionally, S protein itself has been proposed to possess amyloidogenic properties, providing a potential mechanistic explanation for its proposed involvement in amyloid microclot formation [95]. Interestingly, fibrinolysis-resistant microclots have also been described in other chronic inflammatory conditions, including PD [96, 97].

α-Synuclein is an intrinsically disordered, amyloidogenic protein capable of misfolding into amyloid fibrils associated with synucleinopathies such as PD [98, 99]. Although α-synuclein has not been identified as a confirmed component of fibrinaloid microclots, it has been proposed that these structures may entrap different amyloidogenic proteins depending on the underlying disease process. Cross-seeding between different amyloidogenic proteins, including α-synuclein, has been proposed as a potential mechanism contributing to amyloid aggregation and microclot formation [100]. The formation of fibrinaloid microclots is inherently associated with inflammatory processes and may contribute to vascular dysfunction through impaired microcirculatory flow and endothelial injury [101]. It is therefore plausible that the identification of S protein within endothelial structures of capillary vessels by Mörz [20], where microvascular obstruction could theoretically have significant consequences, may be consistent with the presence of S protein-associated microclot structures. Similarly, detection of vaccinal S protein by Brogna et al. could potentially reflect association with fibrinaloid microclot structures, although this remains to be experimentally confirmed [80]. Future studies assessing the relative abundance and composition of human plasma proteins within blood samples used for vaccinal S protein identification, compared with appropriate controls, may help determine whether these samples exhibit molecular features consistent with fibrinaloid microclot architecture.

Assessment: Limited support.

### Coherence

Experimental and clinical literature indicates that SARS-CoV-2 infection and isolated S protein derived from vaccination in model systems, and post-vaccination syndrome involve endothelial dysfunction, immune pathways, and tissue inflammation. The pathology is consistent with previously reported vaccine-related myocarditis and encephalitis [102–107]. This provides limited coherence with the proposed mechanism and does not fully establish that vaccine-encoded S protein caused the lesions in the patient.

Assessment: Limited support.

### Specificity

Although it is one of the weaker criteria in modern epidemiology (as most exposures can have multiple effects, and they may stem from diverse causes), it still offers value when a cause leads to a narrowly defined effect. This criterion was not completely fulfilled since PD was the patient’s underlying diagnosis. The potential contribution of this underlying illness to the encephalitis and myocarditis observed during post-mortem investigation warrants consideration. However, the temporal characteristics clearly distinguish these conditions: PD had been present as a chronic condition for years, whereas the encephalitis represented an acute inflammatory process. On the other hand, no case report of PD causing subsequent necrotizing encephalitis has been reported, nor is there a convincing mechanism. This suggests that the observed encephalitis was unlikely to be a direct consequence of PD [20]. Although the N protein was absent, the patient may have had an asymptomatic infection. Following COVID-19 vaccination, cases of necrotizing encephalitis and encephalomyelitis have been reported [102, 103, 108–114], and numerous studies have reported that SARS-CoV-2 infection can also trigger encephalitis [115–147].

It should be considered that most of these encephalitis cases occurred in 2020, before the initiation of COVID-19 mass vaccination campaigns. This suggests the causal agent was the virus. Of particular interest are five case reports, the first from 2020, on SARS-CoV-2-associated acute haemorrhagic, necrotizing encephalitis presenting with cognitive impairment in a 44-year-old woman without comorbidities [148], and the second, from 2021, reported the case of fatal acute haemorrhagic necrotizing encephalitis in a two-month-old boy [149]. The remaining cases from 2021 reported fatal necrotizing encephalitis associated with COVID-19 [150, 151].

Another important confounding factor is the fact that the official cause of death was aspiration pneumonia. Nevertheless, Mörz reported that the patient experienced a sudden collapse during dinner, notably without coughing or other signs of food aspiration, simply falling from his chair. This opens a debate about whether the sudden loss of consciousness was the result of aspiration pneumonia [20].

Assessment: Limited support.

### Biological gradient (dose-response relationship)

The case report lacks statistical quantification and does not permit assessment of a biological gradient. Nevertheless, the patient received multiple vaccine doses, and each vaccine dose is consistent with a biological gradient. Furthermore, existing reports from peer-reviewed literature have noted: (i) An increased incidence of myocarditis, particularly in young males, after the second dose compared to the first dose of mRNA vaccines [45, 152, 153], (ii) Recent research indicates that individuals with pancreatic cancer who received more COVID-19 booster shots had a worse overall survival rate. Interestingly, the study showed that a poor prognosis for these patients was associated with elevated IgG4 antibody levels brought on by vaccination [154]. Other studies showed different distributions of IgG2 and IgG4 subclasses between BNT162b2 and mRNA-1273 [155] and a higher risk of reinfection in people with more IgG2 or IgG4 [156]. Importantly, population-level dose-specific risks for other outcomes cannot directly be transferred.

Assessment: Limited support.

### Analogy

Analogies can be drawn with other reported cases of vaccine-induced myocarditis and encephalitis [44, 102–107], as well as with the effects of natural infection, which also involve S protein expression and vascular inflammation [81–83]. After Mörz’s work, Mikami et al. [111] later reported a case of an 84-year-old man who died approximately 10 weeks after receiving his fourth COVID-19 vaccination, after being admitted to the hospital due to fever and impaired consciousness. Upon autopsy, the thalamus, pons, and cerebellum showed perivascular T-cell infiltration and acute ischemic change with microhaemorrhage, which were thought to be connected to neurological symptoms. Ascites, pleural fluid accumulation, and right ventricular dilatation all pointed to right heart failure. Despite a negative COVID-19 polymerase chain reaction test, IHC analysis for S and N SARS-CoV-2 antigens was conducted to determine the cause of death. The thalamus, pons, pituitary, and adrenal glands were found to contain just the SARS-CoV-2 S protein. Like Mörz [20], Mikami et al. [111] concluded that the S-positive/N-negative IHC pattern was likely attributable to vaccination rather than viral infection. However, the absence of the N protein is insufficient to definitively confirm an mRNA vaccine-derived origin of the S protein, and the cases cannot be equally compared.

Assessment: Limited support.

### Consistency

Consistency has not been demonstrated. Mörz and Mikami et al. [20, 111] are isolated reports and have not been reproduced in a systematically assembled series using standardized pathology, appropriate comparison groups, blinded assessment, and validated source-specific assays. However, due to the temporal constraints of vaccination, a systematic evaluation may no longer be feasible.

Assessment: Not established.

### Experimental evidence

The IHC findings constitute observational pathological evidence rather than experimental evidence in Hill’s sense. The technique employed does not possess the analytical specificity to distinguish between a naturally occurring S-positive/N-negative pattern and does not adequately rule out an asymptomatic infection. Besides, the case report included positive control stained anti-S1 of the S protein but lacked negative and isotype controls to determine non-specific binding.

Mörz argued that the IHC findings in the report provided direct and compelling evidence that S protein from the vaccine was present in the affected tissues and that this was spatially associated with the lesions. According to that report, his study was the first to provide evidence of vaccine-derived S protein within encephalitic lesions, suggesting that vaccination rather than SARS-CoV-2 infection was the cause of death [20].

However, evaluating whether the detected S protein truly reflects a vaccine origin rather than a natural infection requires considering the underlying dynamics of viral protein turnover in tissues. The balance between production and degradation is reflected in the quantity of proteins [157]. The stability of cellular proteins varies widely, ranging from a few minutes to several hours, and can be strictly controlled in response to a range of pathophysiological inputs, both internal and external [158]. Li et al. [159] employed immunoblot analysis to quantify the half-lives of SARS-CoV-2 proteins in lung epithelial cells, thus considering the dynamic nature of viral protein expression. 0.4 to 8 h was the short half-lives of eighteen of these unstable proteins. With half-lives of more than eight hours, the remaining seven proteins—NSP2, NSP5, NSP10, NSP15, S, N, and M—were comparatively stable. These proteins are degraded by the ubiquitin-proteasome system after the acute infection resolves [159]. These findings strongly suggest that the N protein should remain detectable for 8 h to a few days during acute symptomatic or asymptomatic infections. Therefore, its short half-life cannot be used to infer that its absence demonstrates a vaccine origin. In contrast, the half-life of the S protein is longer.

Coordinated SARS-CoV-2-specific CD4^+^ and CD8^+^ T cell responses are associated with mild disease, and infected cells expressing S protein are cleared by cytotoxic CD8^+^ cells within 1–2 weeks after infection [160]. However, Rong et al. found that long after the virus was cleared, SARS-CoV-2 S protein continued to accumulate in the skull-meninges-brain axis of long COVID patients [161].

Therefore, the definitive method to confirm the presence of vaccine-derived S protein requires amino acid sequence analysis of the S protein itself to verify its unique molecular characteristics. A review of the published literature revealed that only the study by Brogna et al. [80] has conclusively demonstrated the presence of the synthetic S protein in blood. While the mRNA sequences from the BioNTech/Pfizer (BNT162b2) and Moderna (mRNA-1273) vaccines differ, they both direct the synthesis of an identical recombinant S protein. This recombinant S differs from the wild-type S protein due to a double amino acid substitution at residues 986 and 987, replacing lysine and valine with two proline residues ((K986P and V987P), herein designated PP-S). These alterations stabilize the S protein in an inactive prefusion conformation and eliminate a tryptic digestion site, thereby enabling differentiation of vaccine-derived synthetic S protein from naturally occurring S protein in biological fluids via tryptic digestion and mass spectrometry [80].

Trypsin, a hydrolase enzyme, cleaves proteins into smaller polypeptides by targeting peptide bonds adjacent to arginine (R) and lysine (K) residues [162]. This specificity enables differentiation between synthetic and natural S proteins based on their distinct tryptic digestion profiles:

(i) Trypsin digestion of the PP-S protein, encoded by vaccine mRNA, yields a characteristic LD**PP**EAEVQIDR fragment (PP-S marker) [80].
(ii) In contrast, trypsin digestion of the viral S protein generates two smaller fragments: LDK and VEAEVQIDR [80].

This proteomic strategy is specifically designed to identify the source of S protein in tissues or fluids [80, 162]. It can distinguish between the prefusion-stabilized vaccine-encoded S and wild-type viral S [80, 162]. The absence of this mutation in over 6,600,000 sequenced SARS-CoV-2 genomes has shown that none, including the Omicron variant, harbour the K986P and V987P mutations [163], further validating the technique’s reliability in ruling out natural infection as the source of detected S protein.

Ota et al. [164] conducted in situ hybridization (ISH) to determine if S protein expression originated from SARS-CoV-2 virus genetic material or mRNA vaccine genetic source, since some cases displayed positive staining for the S protein via IHC. Such a method was performed for cases of haemorrhagic stroke where the infection history was unclear, or when a considerable period had passed following vaccination. The vaccine and SARS-CoV-2 mRNA were both identified by ISH. This indicates that the vaccine cannot be solely responsible for the spike protein-positive staining seen in patients who have received SARS-CoV-2 vaccination but have no recorded history of viral infection. Notably, the N protein was consistently negative in all cases, confirming the high sensitivity of ISH. This capability allows for the detection of traces of mRNA, which may indicate undetected asymptomatic infections. These results emphasize the importance of being cautious in assuming that the S protein originates exclusively from mRNA vaccination [164]. While Ota et al. [164] detected vaccine mRNA, suggesting possible spike protein synthesis, the simultaneous presence of viral mRNA prevents definitive attribution without protein-level sequencing. There is a possibility that the haemorrhagic stroke was caused by past asymptomatic SARS-CoV-2 infection. Although the N protein was not detected (possibly due to its short half-life), the presence of viral mRNA was detected long after the acute infection had passed. For example, a study found that viral mRNA was identified in 16 (30%) of 53 solid tissue samples collected at 1 month, 38 (27%) of 141 samples collected at 2 months, and 7 (11%) of 66 samples collected at 4 months. 10 distinct solid tissue types (liver, kidney, stomach, intestine, brain, blood vessel, lung, breast, skin, and thyroid) were found to have viral mRNA. Furthermore, 26 (43%) of the 61 solid tissue samples that tested positive for viral RNA also tested positive for subgenomic RNA [165].

Assessment: Not established.

### Strength of association

The strength of association criterion is not met, as it relies on a single case report without a comparison group, incidence estimate, clearly defined proof of vaccine-derived S protein presence in the examined tissues, missing isotype and negative controls. The criterion addresses the statistical strength of the association between an exposure (vaccination) and an outcome (AE). It is often measured using statistical metrics such as the relative risk (RR), the odds ratio (OR), and risk difference. A larger effect size (i.e., a higher RR or OR) suggests a stronger association [166]. The pathological observations may generate a hypothesis, but they cannot quantify whether the outcome occurred more frequently after vaccination than expected or distinguish the proposed effect from background disease and competing causes.

Assessment: Not assessable from a single case.

## Discussion

The qualitative Bradford Hill appraisal of Mörz´s work [20] identifies temporality but limited plausibility, coherence, specificity, biological gradient, analogy, consistency, experimental evidence, and strength of association. This is suggestive but not conclusive evidence for a causal relationship between the effects of the BNT162b2 vaccine and multifocal necrotizing encephalitis and myocarditis. The temporal association is strong; however, being a single-case report, potential confounding by comorbidities (PD and pneumonia), insufficient strength of association, and incomplete experimental data limit the capacity to establish definitive causality. Source attribution is additionally limited because the IHC procedure did not distinguish vaccine-encoded from infection-derived S protein.

The absence of detectable N protein cannot definitively serve as evidence for demonstrating a vaccine origin for S immunoreactivity [164]. Within a Kuhnian vocabulary, the reports by Mörz [20], Mikami et al. [111], and Ota et al. [164] and others may be interpreted as anomalous observations because they raise questions not fully resolved by existing source-attribution methods [15, 20, 111, 164]. They could not specifically detect the presence of the S protein derived from the mRNA vaccine. However, the available evidence does not demonstrate that these reports have produced a Kuhnian “crisis phase”, nor does it establish strong resistance by adherents of a prevailing paradigm even if they constitute an accumulating body of evidence that generates epistemological tension within the scientific community and progressively creates the conditions for revolutionary science and a paradigm shift [15]. Nevertheless, Brogna et al. [80] could have initiated this “crisis phase”, which emerges when a mismatch between empirical evidence and established paradigms becomes increasingly apparent. The same response could reflect uncertainty, methodological limitations, regulatory evidentiary standards, or disagreement regarding the reliability and generalizability of individual case reports. Kuhn therefore supplies one interpretive vocabulary for the case, not evidence of how institutions actually behaved.

At present, the only method capable of distinguishing between the naturally occurring SARS-CoV-2 S protein and vaccine-encoded S protein is the tryptic digestion and mass spectrometry analysis employed by Brogna et al. [80]. Complementary approaches include sequence-specific detection of vaccine mRNA and viral RNA by validated molecular assays [164]. This method is particularly valuable in post-mortem and clinical investigations of SAEs potentially associated with mRNA COVID-19 vaccines but does not enable precise attribution of pathological findings to vaccination rather than viral infection. Additionally, the technique has the potential to enhance the range of methodologies employed in the post-marketing surveillance of mRNA vaccines. This would facilitate a thorough investigation into the molecular mechanisms underpinning rare SAEs, thereby contributing to the optimization of vaccine design and the establishment of robust safety monitoring protocols. Before routine use in pathology, such assays require independent analytical validation in relevant tissues, a predefined detection threshold, blinded assessment, appropriate controls, evaluation of degradation and contamination, and systematic investigation.

Future studies should use this and other techniques for systematically assembled, prespecified autopsy cohorts rather than selected reports of individuals who died after vaccination – with adequate healthy controls, in addition to controlled clinical studies or detailed mechanistic investigations. Works by Mörz [20], Brogna et al. [80], Mikami et al. [111], Choi et al. [104], Sung et al. [83], Ota et al. [164], and more systematic approaches by Hulcher et al. [44] exemplify the epistemological constraints that may limit pathological interpretation when empirical findings challenge established paradigmatic frameworks, potentially resulting in systematic underappreciation of novel causal mechanisms. The molecular differentiation [80] between vaccine-induced and infection-induced S protein represents a paradigmatically disruptive innovation that provides empirical evidence for vaccine-related pathology, previously attributed to coincidental occurrence or alternative aetiologies. As booster vaccinations continue to be promoted, it is urgent to implement the recommended strategies in epidemiological studies to estimate whether the pathological outcome occurs more frequently after vaccination than expected. Mörz and Mikami et al. provide hypothesis-generating case observations; Brogna et al. provide a proposed analytical method; and Ota et al. illustrate both the potential value and the limitations of tissue-based molecular source attribution [20, 80, 111, 164]. These studies are complementary, but they cannot establish proof of vaccine-related pathology.

Recently, Mörz et al. published another case report of two deceased patients (one with myocarditis, one with hepatitis) who had received COVID-19 vaccines. Using IHC again, the authors continued to argue that the S-positive/N-negative IHC pattern was likely attributable to vaccination rather than viral infection, effectively ruling out a natural viral infection in the affected organs [167]. Notably, they reached that conclusion through an indirect, exclusionary argument rather than a direct confirmatory one. It is therefore imperative that tissue samples from the individuals who died in the studies mentioned above be re-examined using mass spectrometry, as described by Brogna et al. [80], which is currently the only one that can definitively establish whether the S protein identified is of viral origin or derived from the mRNA vaccine.

Kuhn’s framework is particularly relevant to the past COVID-19 pandemic and future pandemics, where the rapid development of novel vaccine technologies coincided with intense pressure to maintain public trust and policy coherence. In this context, any data that challenged the dominant paradigm that vaccines are inherently safe, effective, and largely free of SAEs was at risk of being treated not as anomalies warranting further study but as threats to be neutralized. Statements such as ‘the vaccine remains localized at the injection site’ and ‘the S protein is rapidly degraded’ served to reinforce the dominant narrative by providing reassurance. Yet emerging data refuted these claims, e.g., [168]. For instance, biodistribution studies have documented S protein expression both as a membrane-bound antigen on transfected cells and as a soluble protein detected in the blood circulation [80, 169], indicating systemic antigen dissemination beyond the injection site. Several works have reported that the mRNA vaccines (or their components) can travel to distant organs, including the liver [169, 170], lungs [169, 171], kidneys [169, 171], lymph nodes [172–174], spleen [169, 171], heart [169, 174], and brain [169, 175]. Still, they seem to be independent epidemiological and mechanistic events.

Brogna et al. reported vaccine-encoded S-protein peptides in selected blood samples 69 and 187 days post vaccination [80]. Ota et al. found S protein immunoreactivity in cerebral arteries in patients with haemorrhagic stroke up to 17 months after vaccination, suggesting potential long-term tissue persistence [164]. These findings raise questions about persistence and biodistribution of mRNA vaccines and their long-term safety, as their discovery finding that 43.8% (7 out of 16) of vaccinated individuals had S protein immunoreactivity [164]. However, not all of these cases necessarily had S protein derived exclusively from the vaccine. ISH was conducted on three of these seven cases, and it detected both the vaccine-derived mRNA and SARS-CoV-2 viral mRNA, indicating a mixed origin of the S protein expression. The three cases (4, 5, and 15) represented 18.75% of the total 16 vaccinated individuals in the study. The authors acknowledged that asymptomatic SARS-CoV-2 infection could be a possible factor [164].

This finding further complicates the determination of causality, as the observed haemorrhagic stroke cases may result from the combined effects of both viral and synthetic spike proteins and remain hypotheses requiring independent epidemiological and mechanistic testing. Recent work has proposed that SAEs associated with the COVID-19 mRNA vaccines might be amplified by SARS-CoV-2 infection. The authors have coined the “Hybrid Harm Hypothesis”, which posits that “interactions between COVID-19 mRNA injections and later coronavirus infections may explain the manifestation and/or persistence of SAEs in previously vaccinated individuals, even after the emergence of milder Omicron variants. In many cases, the biological impact of COVID-19 mRNA vaccination may constitute a precursor event, predisposing the individual to develop the post-COVID-19 sequelae. Coronavirus infections may amplify the adverse effects of previous COVID-19 vaccinations over the course of years rather than months” [176].

According to Patterson et al., the S protein subunit S1, which is associated with inflammation and symptoms resembling those of post-acute COVID-19 sequelae, can remain in some monocytes for up to 245 days following vaccination [177]. This suggests that some individuals may experience prolonged symptoms potentially attributable to the persistence of S protein [168, 177]. A recent preprint found that two out of 40 individuals with post-vaccination syndrome (PVS) had measurable S protein levels for over 700 days after vaccination; however, this was not consistent for all PVS patients. The majority of those with detectable S protein levels had it in the lower range (26–300 days). Although additional research is required, this prolonged persistence may be linked to chronic symptoms [178]. Mechanistic differences between natural SARS-CoV-2 infection and mRNA vaccination should also be considered. During infection, Fcγ receptor-mediated uptake of virus into monocytes results in abortive infection and infectious virus is not detected in infected monocytes in cell culture because infected cells undergo pyroptosis but this process causes systemic inflammation that contributes to COVID-19 pathology [179]. However, these two studies were unable to differentiate between the SARS-CoV-2 S protein and the synthetic one, so they cannot be used to draw causal inferences in forensic pathology.

The central claim of this article is normative rather than demonstrative: the absence of established causality should not automatically be treated as a reason to terminate investigations of a credible safety signal. It is not sufficient to report all safety signals occurring after vaccination only as “coincidental” without adequate investigation and to use the principle “correlation does not imply causality” repeatedly. At the same time, temporal proximity, pathological co-localization, or detection of an exposure-related molecule does not independently establish causation. Mörz [20] illustrates this evidentiary tension but cannot support a broader conclusion that vaccine-safety research was systematically obstructed. The history of science reminds us that progress does not come from the passive accumulation of data and the critical evaluation of correlations for causal meaning. Kuhn’s framework helps articulate how anomalous observations may be interpreted within established scientific frameworks, whereas sociology of science identifies institutional conditions that could have affected the reception of dissenting claims. Both can blind scientists to anomalies and inhibit exploration of alternative explanations. Whether these mechanisms operated systematically during the COVID-19 pandemic remains an empirical question; we can only take circumstantial evidence into account. Addressing it would require systematic analysis of institutional communications and research practices, alongside controlled, epidemiological, pathological, and molecular studies. Some of this work is already done. Much of science advances precisely by uncovering correlations that are later shown to be either causative or, more often, spurious. But if causality is not actively investigated, science risks falling into fallacies that not only erode the integrity of research but also jeopardize the well-being of populations who rely on it. True scientific progress therefore requires both a willingness to investigate anomalous findings and an equal willingness to reject causal claims when the evidence remains insufficient.

Despite the favourable benefit-risk profile of anti-SARS-CoV-2 mRNA vaccines for elderly individuals and those with age-related or other comorbidities and the potential of mRNA vaccines against other infectious disease prevention and public health preparedness, and for the treatment of cancer and autoimmunity, a comprehensive investigation into the molecular and cellular mechanisms underlying vaccine-induced AEs is mandatory for the continued success of vaccination and booster campaigns. Concurrently, the prospective pharmacovigilance and long-term monitoring of vaccinated individuals in comparison to matched controls should be conducted in well-designed clinical trials [180, 181]. An important ethical question remains whether describing severe adverse events as “rare” is sufficient from the perspective of those individuals and families who experience irreversible harm.

## Data Availability

No datasets were generated or analysed during the current study.
